# Diaqua­bis­[*N*-(pyridin-4-yl)isonicotin­amide-κ*N*]bis­(thio­cyanato-κ*N*)cobalt(II)

**DOI:** 10.1107/S1600536812024105

**Published:** 2012-06-02

**Authors:** Jacob W. Uebler, Robert L. LaDuca

**Affiliations:** a Lyman Briggs College, Department of Chemistry, Michigan State University, East Lansing, MI 48825, USA

## Abstract

In the title compound, [Co(NCS)_2_(C_11_H_9_N_3_O)_2_(H_2_O)_2_], the octa­hedrally coordinated Co^II^ ion lies on a crystallographic inversion center and is bound by two isothio­cyanate ligands, two aqua ligands and two *N*-(pyridin-4-yl)isonicotinamide (4-pina) ligands. The dihedral angle between the aromatic rings in the 4-pina ligand is 8.98 (11)°. In the crystal, the individual mol­ecular units are aggregated in three dimensions by O—H⋯N, O—H⋯S and N—H⋯S hydrogen-bonding pathways.

## Related literature

For other cobalt isothio­cyanate coordination polymers containing dipyridyl ligands, see: Johnston *et al.* (2007 ▶); Martin *et al.* (2009 ▶). For other coordination polymers containing the 4-pina ligand, see: Uemura *et al.* (2008 ▶). For the synthesis of the 4-pina ligand, see: Gardner *et al.* (1954) ▶.
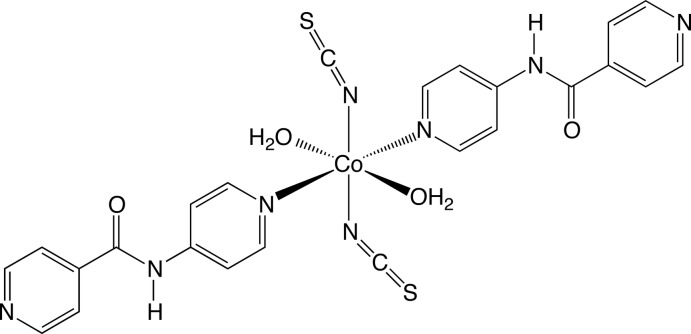


## Experimental

### 

#### Crystal data

[Co(NCS)_2_(C_11_H_9_N_3_O)_2_(H_2_O)_2_]
*M* *_r_* = 609.55
Triclinic, 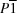

*a* = 7.0651 (4) Å
*b* = 9.3943 (5) Å
*c* = 10.5943 (6) Å
α = 81.433 (1)°
β = 76.343 (1)°
γ = 71.697 (1)°
*V* = 646.58 (6) Å^3^
*Z* = 1
Mo *K*α radiation
μ = 0.87 mm^−1^
*T* = 173 K
0.30 × 0.19 × 0.16 mm

#### Data collection

Bruker APEXII CCD diffractometer
Absorption correction: multi-scan (*SADABS*; Sheldrick, 1996 ▶) *T*_min_ = 0.778, *T*_max_ = 0.873
10537 measured reflections
2348 independent reflections
2224 reflections with *I* > 2σ(*I*)
*R*_int_ = 0.021

#### Refinement

*R*[*F* ^2^ > 2σ(*F* ^2^)] = 0.031
*wR*(*F* ^2^) = 0.079
*S* = 1.06
2348 reflections
187 parameters
4 restraints
H atoms treated by a mixture of independent and constrained refinement
Δρ_max_ = 1.07 e Å^−3^
Δρ_min_ = −0.33 e Å^−3^

### 

Data collection: *APEX2* (Bruker, 2006 ▶); cell refinement: *SAINT* (Bruker, 2006 ▶); data reduction: *SAINT*; program(s) used to solve structure: *SHELXS97* (Sheldrick, 2008 ▶); program(s) used to refine structure: *SHELXL97* (Sheldrick, 2008 ▶); molecular graphics: *SHELXTL* (Sheldrick, 2008 ▶); software used to prepare material for publication: *SHELXTL*.

## Supplementary Material

Crystal structure: contains datablock(s) I, global. DOI: 10.1107/S1600536812024105/hb6817sup1.cif

Structure factors: contains datablock(s) I. DOI: 10.1107/S1600536812024105/hb6817Isup2.hkl

Additional supplementary materials:  crystallographic information; 3D view; checkCIF report

## Figures and Tables

**Table 1:** Selected bond lengths (Å)

Co1—O1	2.0964 (15)
Co1—N4	2.0994 (18)
Co1—N1	2.1410 (16)

**Table 2:** Hydrogen-bond geometry (Å, °)

*D*—H⋯*A*	*D*—H	H⋯*A*	*D*⋯*A*	*D*—H⋯*A*
O1—H1*A*⋯S1^i^	0.83 (2)	2.52 (2)	3.3129 (16)	162 (2)
O1—H1*B*⋯N3^ii^	0.84 (2)	1.95 (2)	2.755 (2)	162 (2)
N2—H2*N*⋯S1^iii^	0.93 (2)	2.68 (2)	3.540 (2)	155 (2)

Symmetry codes: (i) 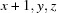; (ii) 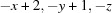; (iii) 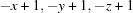.

**Fig. 1.**
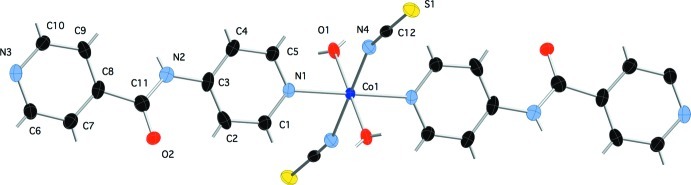
The coordination environment of the title compound, showing 50% probability ellipsoids and partial atom numbering scheme. Unlabelled atoms are generated by (1–x, –y, 1–z). Hydrogen atom positions are shown as grey sticks. Color codes: dark blue Co, red O, black C, light blue N, yellow S.

**Fig. 2.**
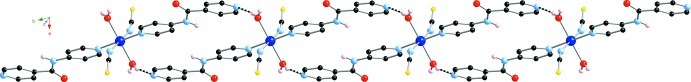
A supramolecular chain of [Co(H2O)2(NCS)2(4-pina)2] molecules formed by O—H···N hydrogen bonding, which is indicated as dashed lines.

**Fig. 3.**
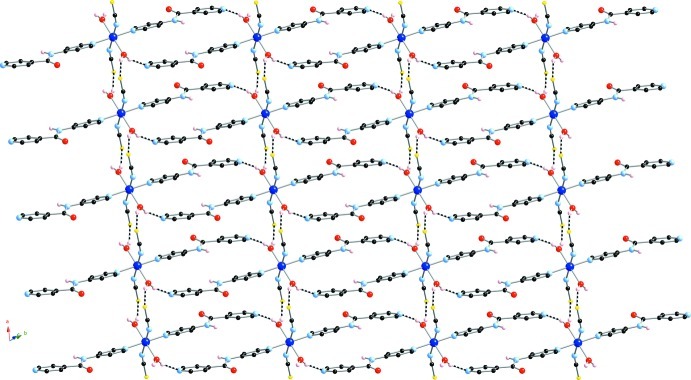
A supramolecular layer of chains of [Co(H2O)2(NCS)2(4-pina)2] molecules formed by O—H···S hydrogen bonding, which is indicated as dashed lines.

**Fig. 4.**
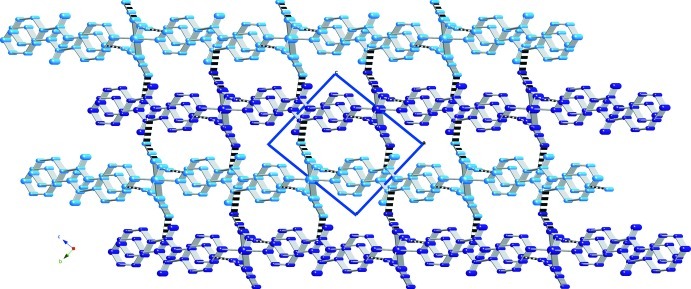
Stacking diagram of the title compound. Supramolecular layers are aggregated by by N—H···S hydrogen bonding, which is indicated as dashed lines.

## supplementary crystallographic information

### Comment

In an attempt to prepare cobalt isothiocyanato coordination polymers containing 4-pyridylisonicotinamide (4-pina), the title compound, [Co(H_2_O)_2_(NCS)_2_(C_11_H_9_N_3_O)_2_], was isolated.

The asymmetric unit of the title compound contains a Co^II^ ion on a crystallographic inversion center, one aqua ligand, one N-bound isothiocyanato ligand, and one 4-pina ligand bound *via* the pyridyl ring closest to the amide N atom. Operation of the inversion center produces a complete [Co(H_2_O)_2_(NCS)_2_(4-pina)_2_] molecular complex (Fig. 1). The Co^II^ ion is octahedrally coordinated with *trans* aqua ligands, *trans* isothiocyanato ligands and *trans* 4-pina ligands. One of the pyridyl termini of the 4-pina ligand remains unligated and unprotonated.

Individual [Co(H_2_O)_2_(NCS)_2_(4-pina)_2_] complexes are connected into supramolecular chain motifs oriented along the [1 1 0] crystal direction (Fig. 2) *via* O—H···N hydrogen bonding between aqua ligands and unligated pyridyl N atoms. In turn these supramolecular chains aggregate into layer motifs (Fig. 3) by means of O—H···S hydrogen bonding between aqua ligands and terminal S atoms of the isothiocyanato ligands. These layers are coincident with the crystallographic (0 1 1) planes. These planes further aggregate into the three-dimensional crystal structure of the title compound (Fig. 4) through N—H···S hydrogen bonding between amide N—H groups of the 4-pina ligands and terminal S atoms of the isothiocyanato ligands.

### Experimental

Cobalt(II) thiocyanate was obtained commercially. 4-Pyridylisonicotinamide was prepared by a published procedure (Gardner *et al.*, 1954). Cobalt(II) thiocyanate (23 mg, 0.13 mmol) was dissolved in 3 ml water in a 15 ml glass vial. Onto this solution was layered 2 ml of a 1:1 water:ethanol solution, followed by a solution of 4-pina (19 mg, 0.10 mmol) dissolved in 3 ml 95% ethanol. Pink blocks of the title compound (16 mg, 0.026 mmol, 55% yield based on 4-pina) were obtained after 14 d at 298 K, and were isolated after washing with distilled water and acetone, and drying in air.

### Refinement

All H atoms bound to C atoms were placed in calculated positions, with C—H = 0.95 Å, and refined in riding mode with *U*_iso_ = 1.2*U*_eq_(C). The H atoms bound to the aqua ligand O atom were found in a difference Fourier map, restrained with with O—H = 0.85 Å and refined with *U*_iso_ = 1.2*U*_eq_(O). The H atom bound to the 4-pina ligand N atom was found in a difference Fourier map, restrained with with N—H = 0.90 Å and refined with *U*_iso_ = 1.2*U*_eq_(N).

### Crystal data

**Table:**

[Co(NCS)_2_(C_11_H_9_N_3_O)_2_(H_2_O)_2_]	*Z* = 1
*M**_r_* = 609.55	*F*(000) = 313
Triclinic, *P*1	*D*_x_ = 1.565 Mg m^−^^3^
*a* = 7.0651 (4) Å	Mo *K*α radiation, λ = 0.71073 Å
*b* = 9.3943 (5) Å	Cell parameters from 7965 reflections
*c* = 10.5943 (6) Å	θ = 2.3–25.3°
α = 81.433 (1)°	µ = 0.87 mm^−^^1^
β = 76.343 (1)°	*T* = 173 K
γ = 71.697 (1)°	Prism, pink
*V* = 646.58 (6) Å^3^	0.30 × 0.19 × 0.16 mm

### Data collection

**Table:**

Bruker APEXII CCD diffractometer	2348 independent reflections
Radiation source: fine-focus sealed tube	2224 reflections with *I* > 2σ(*I*)
Graphite monochromator	*R*_int_ = 0.021
φ and ω scans	θ_max_ = 25.3°, θ_min_ = 2.0°
Absorption correction: multi-scan (*SADABS*; Sheldrick, 1996)	*h* = −8→8
*T*_min_ = 0.778, *T*_max_ = 0.873	*k* = −11→11
10537 measured reflections	*l* = −12→12

### Refinement

**Table:**

Refinement on *F*^2^	Primary atom site location: structure-invariant direct methods
Least-squares matrix: full	Secondary atom site location: difference Fourier map
*R*[*F*^2^ > 2σ(*F*^2^)] = 0.031	Hydrogen site location: inferred from neighbouring sites
*wR*(*F*^2^) = 0.079	H atoms treated by a mixture of independent and constrained refinement
*S* = 1.06	*w* = 1/[σ^2^(*F*_o_^2^) + (0.0374*P*)^2^ + 0.6702*P*] where *P* = (*F*_o_^2^ + 2*F*_c_^2^)/3
2348 reflections	(Δ/σ)_max_ < 0.001
187 parameters	Δρ_max_ = 1.07 e Å^−^^3^
4 restraints	Δρ_min_ = −0.33 e Å^−^^3^

### Special details

**Table:**

Experimental. REM Highest difference peak 1.065, 1.00 Å from N2
Geometry. All e.s.d.'s (except the e.s.d. in the dihedral angle between two l.s. planes) are estimated using the full covariance matrix. The cell e.s.d.'s are taken into account individually in the estimation of e.s.d.'s in distances, angles and torsion angles; correlations between e.s.d.'s in cell parameters are only used when they are defined by crystal symmetry. An approximate (isotropic) treatment of cell e.s.d.'s is used for estimating e.s.d.'s involving l.s. planes.
Refinement. Refinement of *F*^2^ against ALL reflections. The weighted *R*-factor *wR* and goodness of fit *S* are based on *F*^2^, conventional *R*-factors *R* are based on *F*, with *F* set to zero for negative *F*^2^. The threshold expression of *F*^2^ > σ(*F*^2^) is used only for calculating *R*-factors(gt) *etc*. and is not relevant to the choice of reflections for refinement. *R*-factors based on *F*^2^ are statistically about twice as large as those based on *F*, and *R*- factors based on ALL data will be even larger.

### Fractional atomic coordinates and isotropic or equivalent isotropic displacement parameters (Å^2^)

**Table:**

	*x*	*y*	*z*	*U*_iso_*/*U*_eq_	
Co1	0.5000	1.0000	0.5000	0.01560 (12)	
S1	0.01686 (8)	0.77868 (6)	0.79444 (5)	0.02645 (15)	
O1	0.7779 (2)	0.86741 (18)	0.54694 (15)	0.0256 (3)	
H1A	0.815 (4)	0.861 (3)	0.6164 (18)	0.031*	
H1B	0.879 (3)	0.847 (3)	0.4857 (19)	0.031*	
O2	0.5678 (2)	0.71752 (16)	−0.10588 (14)	0.0266 (3)	
N1	0.5585 (3)	0.86061 (19)	0.34441 (16)	0.0198 (4)	
N2	0.7178 (3)	0.5618 (2)	0.05144 (18)	0.0283 (4)	
H2N	0.790 (4)	0.462 (2)	0.066 (2)	0.034*	
N3	0.9194 (3)	0.2524 (2)	−0.34165 (17)	0.0246 (4)	
N4	0.3440 (3)	0.8620 (2)	0.62833 (17)	0.0247 (4)	
C1	0.5605 (3)	0.9145 (2)	0.2200 (2)	0.0246 (5)	
H1	0.5281	1.0203	0.2002	0.030*	
C2	0.6077 (4)	0.8233 (3)	0.1186 (2)	0.0301 (5)	
H2	0.6058	0.8663	0.0317	0.036*	
C3	0.6578 (3)	0.6679 (3)	0.1461 (2)	0.0263 (5)	
C4	0.6567 (3)	0.6116 (3)	0.2740 (2)	0.0280 (5)	
H4	0.6907	0.5062	0.2965	0.034*	
C5	0.6054 (3)	0.7106 (2)	0.3685 (2)	0.0248 (5)	
H5	0.6031	0.6702	0.4564	0.030*	
C6	0.8466 (3)	0.3991 (3)	−0.3756 (2)	0.0273 (5)	
H6	0.8510	0.4297	−0.4656	0.033*	
C7	0.7661 (3)	0.5084 (3)	−0.2891 (2)	0.0273 (5)	
H7	0.7144	0.6110	−0.3187	0.033*	
C8	0.7621 (3)	0.4658 (2)	−0.1579 (2)	0.0243 (5)	
C9	0.8341 (3)	0.3146 (3)	−0.1199 (2)	0.0270 (5)	
H9	0.8317	0.2813	−0.0305	0.032*	
C10	0.9102 (3)	0.2119 (2)	−0.2148 (2)	0.0261 (5)	
H10	0.9578	0.1081	−0.1879	0.031*	
C11	0.6720 (3)	0.5941 (3)	−0.0696 (2)	0.0274 (5)	
C12	0.2111 (3)	0.8259 (2)	0.69719 (19)	0.0200 (4)	

### Atomic displacement parameters (Å^2^)

**Table:**

	*U*^11^	*U*^22^	*U*^33^	*U*^12^	*U*^13^	*U*^23^
Co1	0.0173 (2)	0.0152 (2)	0.0135 (2)	−0.00330 (15)	−0.00210 (14)	−0.00348 (14)
S1	0.0252 (3)	0.0293 (3)	0.0243 (3)	−0.0109 (2)	−0.0049 (2)	0.0058 (2)
O1	0.0213 (8)	0.0345 (9)	0.0165 (7)	0.0012 (7)	−0.0044 (6)	−0.0072 (6)
O2	0.0319 (8)	0.0210 (8)	0.0237 (8)	0.0009 (6)	−0.0110 (6)	−0.0021 (6)
N1	0.0195 (8)	0.0203 (8)	0.0191 (8)	−0.0032 (7)	−0.0039 (7)	−0.0055 (7)
N2	0.0317 (10)	0.0254 (10)	0.0266 (10)	−0.0055 (8)	−0.0058 (8)	−0.0045 (8)
N3	0.0220 (9)	0.0267 (9)	0.0241 (9)	−0.0042 (7)	−0.0014 (7)	−0.0105 (7)
N4	0.0288 (10)	0.0241 (9)	0.0212 (9)	−0.0094 (8)	−0.0028 (8)	−0.0019 (7)
C1	0.0270 (11)	0.0245 (11)	0.0223 (11)	−0.0054 (9)	−0.0064 (9)	−0.0043 (8)
C2	0.0321 (12)	0.0437 (14)	0.0167 (10)	−0.0123 (10)	−0.0050 (9)	−0.0057 (9)
C3	0.0195 (10)	0.0301 (12)	0.0317 (12)	−0.0087 (9)	0.0005 (9)	−0.0160 (9)
C4	0.0282 (11)	0.0225 (11)	0.0324 (12)	−0.0051 (9)	−0.0029 (9)	−0.0097 (9)
C5	0.0271 (11)	0.0224 (11)	0.0245 (11)	−0.0051 (9)	−0.0053 (9)	−0.0057 (9)
C6	0.0287 (12)	0.0295 (12)	0.0220 (11)	−0.0059 (9)	−0.0032 (9)	−0.0058 (9)
C7	0.0270 (11)	0.0232 (11)	0.0308 (12)	−0.0064 (9)	−0.0030 (9)	−0.0058 (9)
C8	0.0173 (10)	0.0274 (11)	0.0306 (11)	−0.0098 (9)	0.0010 (8)	−0.0122 (9)
C9	0.0264 (11)	0.0387 (13)	0.0179 (10)	−0.0121 (10)	−0.0025 (8)	−0.0059 (9)
C10	0.0261 (11)	0.0228 (11)	0.0279 (11)	−0.0039 (9)	−0.0051 (9)	−0.0048 (9)
C11	0.0247 (11)	0.0328 (13)	0.0265 (11)	−0.0130 (10)	−0.0024 (9)	−0.0027 (9)
C12	0.0246 (11)	0.0158 (9)	0.0195 (10)	−0.0030 (8)	−0.0081 (9)	−0.0016 (8)

### Geometric parameters (Å, º)

**Table:**

Co1—O1	2.0964 (15)	C1—C2	1.388 (3)
Co1—O1^i^	2.0964 (15)	C1—H1	0.9500
Co1—N4^i^	2.0994 (18)	C2—C3	1.392 (3)
Co1—N4	2.0994 (18)	C2—H2	0.9500
Co1—N1^i^	2.1410 (16)	C3—C4	1.379 (3)
Co1—N1	2.1410 (16)	C4—C5	1.376 (3)
S1—C12	1.649 (2)	C4—H4	0.9500
O1—H1A	0.825 (16)	C5—H5	0.9500
O1—H1B	0.836 (16)	C6—C7	1.373 (3)
O2—C11	1.225 (3)	C6—H6	0.9500
N1—C1	1.338 (3)	C7—C8	1.384 (3)
N1—C5	1.342 (3)	C7—H7	0.9500
N2—C11	1.364 (3)	C8—C9	1.383 (3)
N2—C3	1.418 (3)	C8—C11	1.518 (3)
N2—H2N	0.927 (17)	C9—C10	1.392 (3)
N3—C10	1.332 (3)	C9—H9	0.9500
N3—C6	1.337 (3)	C10—H10	0.9500
N4—C12	1.154 (3)		
			
O1—Co1—O1^i^	180.0	C1—C2—H2	120.5
O1—Co1—N4^i^	88.84 (7)	C3—C2—H2	120.5
O1^i^—Co1—N4^i^	91.16 (7)	C4—C3—C2	118.11 (19)
O1—Co1—N4	91.16 (7)	C4—C3—N2	117.0 (2)
O1^i^—Co1—N4	88.84 (7)	C2—C3—N2	124.9 (2)
N4^i^—Co1—N4	180.0	C5—C4—C3	118.9 (2)
O1—Co1—N1^i^	91.88 (6)	C5—C4—H4	120.6
O1^i^—Co1—N1^i^	88.12 (6)	C3—C4—H4	120.6
N4^i^—Co1—N1^i^	91.27 (7)	N1—C5—C4	124.2 (2)
N4—Co1—N1^i^	88.73 (7)	N1—C5—H5	117.9
O1—Co1—N1	88.12 (6)	C4—C5—H5	117.9
O1^i^—Co1—N1	91.88 (6)	N3—C6—C7	124.4 (2)
N4^i^—Co1—N1	88.73 (7)	N3—C6—H6	117.8
N4—Co1—N1	91.27 (7)	C7—C6—H6	117.8
N1^i^—Co1—N1	180.0	C6—C7—C8	118.5 (2)
Co1—O1—H1A	127.1 (18)	C6—C7—H7	120.8
Co1—O1—H1B	117.5 (17)	C8—C7—H7	120.8
H1A—O1—H1B	110 (2)	C9—C8—C7	118.34 (19)
C1—N1—C5	116.63 (18)	C9—C8—C11	126.7 (2)
C1—N1—Co1	123.33 (14)	C7—C8—C11	114.90 (19)
C5—N1—Co1	119.97 (14)	C8—C9—C10	118.9 (2)
C11—N2—C3	124.0 (2)	C8—C9—H9	120.6
C11—N2—H2N	112.7 (16)	C10—C9—H9	120.6
C3—N2—H2N	123.3 (16)	N3—C10—C9	123.2 (2)
C10—N3—C6	116.73 (18)	N3—C10—H10	118.4
C12—N4—Co1	159.23 (17)	C9—C10—H10	118.4
N1—C1—C2	123.2 (2)	O2—C11—N2	123.0 (2)
N1—C1—H1	118.4	O2—C11—C8	121.9 (2)
C2—C1—H1	118.4	N2—C11—C8	115.1 (2)
C1—C2—C3	119.0 (2)	N4—C12—S1	178.42 (19)
			
O1—Co1—N1—C1	−124.58 (17)	C11—N2—C3—C2	−22.5 (3)
O1^i^—Co1—N1—C1	55.42 (17)	C2—C3—C4—C5	0.3 (3)
N4^i^—Co1—N1—C1	−35.70 (17)	N2—C3—C4—C5	178.0 (2)
N4—Co1—N1—C1	144.30 (17)	C1—N1—C5—C4	0.8 (3)
N1^i^—Co1—N1—C1	27 (35)	Co1—N1—C5—C4	−176.15 (17)
O1—Co1—N1—C5	52.17 (16)	C3—C4—C5—N1	−1.0 (3)
O1^i^—Co1—N1—C5	−127.83 (16)	C10—N3—C6—C7	0.5 (3)
N4^i^—Co1—N1—C5	141.05 (16)	N3—C6—C7—C8	1.1 (3)
N4—Co1—N1—C5	−38.95 (16)	C6—C7—C8—C9	−1.8 (3)
N1^i^—Co1—N1—C5	−156 (35)	C6—C7—C8—C11	179.56 (19)
O1—Co1—N4—C12	156.5 (5)	C7—C8—C9—C10	0.9 (3)
O1^i^—Co1—N4—C12	−23.5 (5)	C11—C8—C9—C10	179.4 (2)
N4^i^—Co1—N4—C12	−129 (100)	C6—N3—C10—C9	−1.5 (3)
N1^i^—Co1—N4—C12	64.7 (5)	C8—C9—C10—N3	0.8 (3)
N1—Co1—N4—C12	−115.3 (5)	C3—N2—C11—O2	−2.7 (3)
C5—N1—C1—C2	0.1 (3)	C3—N2—C11—C8	176.58 (19)
Co1—N1—C1—C2	176.94 (16)	C9—C8—C11—O2	−161.6 (2)
N1—C1—C2—C3	−0.7 (3)	C7—C8—C11—O2	16.8 (3)
C1—C2—C3—C4	0.5 (3)	C9—C8—C11—N2	19.1 (3)
C1—C2—C3—N2	−177.0 (2)	C7—C8—C11—N2	−162.41 (19)
C11—N2—C3—C4	159.9 (2)	Co1—N4—C12—S1	7 (8)

Symmetry code: (i) −*x*+1, −*y*+2, −*z*+1.

### Hydrogen-bond geometry (Å, º)

**Table:**

*D*—H···*A*	*D*—H	H···*A*	*D*···*A*	*D*—H···*A*
O1—H1*A*···S1^ii^	0.83 (2)	2.52 (2)	3.3129 (16)	162 (2)
O1—H1*B*···N3^iii^	0.84 (2)	1.95 (2)	2.755 (2)	162 (2)
N2—H2*N*···S1^iv^	0.93 (2)	2.68 (2)	3.540 (2)	155 (2)

Symmetry codes: (ii) *x*+1, *y*, *z*; (iii) −*x*+2, −*y*+1, −*z*; (iv) −*x*+1, −*y*+1, −*z*+1.
